# Characterization of Thyroid Hormones Antivertigo Effects in a Rat Model of Excitotoxically-Induced Vestibulopathy

**DOI:** 10.3389/fneur.2022.877319

**Published:** 2022-05-25

**Authors:** Claire M. Bringuier, Bérenice Hatat, Romain Boularand, Christian Chabbert, Brahim Tighilet

**Affiliations:** ^1^Vertidiag, Montpellier, France; ^2^Aix Marseille Université-CNRS, Laboratoire de Neurosciences Cognitives, LNC UMR 7291, Marseille, France

**Keywords:** thyroxine, triiodothyronine, vertigo, vestibulopathy, vestibular disorder, behavior, rat

## Abstract

Impaired vestibular function induces disabling symptoms such as postural imbalance, impaired locomotion, vestibulo-ocular reflex alteration, impaired cognitive functions such as spatial disorientation, and vegetative deficits. These symptoms show up in sudden attacks in patients with Ménière or neuritis and may lead to emergency hospitalizations. To date, however, there is no curative solution to these pathologies and the effectiveness of treatments used to reduce symptoms in the management of patients is discussed. Thus, elucidating the biological mechanisms correlated to the expression kinetics of the vestibular syndrome is useful for the development of potential therapeutic candidates with a view to relieving patients and limiting emergency hospitalizations. Recently, a robust antivertigo effect of thyroxine (T4) was demonstrated in a rodent model of impaired vestibular function induced by unilateral surgical section of the vestibular nerve. The aim of the present study was to assess thyroid hormones L-T4 and triiodothyronine (T3) as well as the bioactive thyroid hormone metabolite TRIAC on a rodent model of acute unilateral vestibulopathy more representative of clinical vestibular pathology. To this end, a partial and transient unilateral suppression of peripheral vestibular inputs was induced by an excitotoxic lesion caused by transtympanic injection of kainic acid (TTK) into the inner ear of adult rats. Vestibular syndrome and functional recovery were studied by semi-quantitative and quantitative assessments of relevant posturo-locomotor parameters. In contrast to the effect previously demonstrated in the complete and irreversible vestibular injury model, administration of thyroxine in the TTK rodent model did not display significant antivertigo effect. However, it is noteworthy that administration of thyroxine showed trends to prevent posturo-locomotor alterations. Furthermore, the results of the current study suggested that a single dose of thyroxine is sufficient to induce the same effects on vestibular syndrome observed with sub-chronic administration, and that reducing the T4 dose may more efficiently prevent the appearance of vestibular deficits induced by the excitotoxic type lesion. Finally, comparison of the antivertigo effect of T4 in different vestibulopathy models enables us to determine the therapeutic indication in which thyroxine could be a potential therapeutic candidate.

## Introduction

Many vestibular disorders are characterized by sudden episodes of functional alterations including a loss of postural balance, impaired locomotion, spatial disorientation, deficits in vestibulo-ocular reflexes, expression of a nystagmus and alteration of vegetative and cognitive functions (1). Symptoms regress gradually and spontaneously and this phenomenon is known as vestibular compensation (2–5). Vestibular disorders represent 3% of all medical prescriptions in the population aged over 50 (6) and nearly 1% of hospital emergencies (7). To date, however, there is no curative solution to these pathologies and the effectiveness of current treatments used to alleviate vestibular symptoms is discussed. To meet this therapeutic need, animal models of vestibular pathologies have been developed to extend the study of the underlying biological mechanisms and to identify new potential therapeutic candidates.

The antivertigo effect of levothyroxine (L-T4) was recently demonstrated in a rodent model of vestibulopathy induced by unilateral vestibular neurectomy (UVN) (8). In this model, acute L-T4 treatment accelerates the recovery of postural and locomotor functions in UVN rats. T4, like triiodothyronine (T3), is produced and secreted endogenously by the thyroid gland, under the control of hypothalamic-pituitary-thyroid axis and subjected to negative feedback when blood levels of T4 and T3 are sufficiently high. T4 acts as a pro-hormone which is converted to active T3 by type 2 deiodase (DIO2). At the cellular level, the presence of thyroid hormone receptors (TRα and TRβ) and the converting enzyme DIO2 has been demonstrated for the first time in the vestibular nuclei (9), confirming in these nuclei, the presence of cellular targets for thyroid hormones.

The present study investigates the effect of thyroid hormones L-T4 and T3 as well as the bioactive thyroid hormone metabolite TRIAC on vestibular disorder in a context of preclinical study. This study was carried out on a previously characterized rodent excitotoxic vestibulopathy model (10–12). Unilateral transtympanic injection of kainic acid (TTK) causes transient and partial deafferentation of vestibular nerve fibers that contact hair cells in the inner ear. The TTK model reproduces symptoms similar in expression and kinetics to those found in certain vestibulopathies such as Ménière's disease.

The aim of this study is first to assess the effect of T4 treatment on a rodent model of vestibulopathy (TTK) close to vestibular pathology, then to determine the optimum dose and window of administration for treating the syndrome. Secondly, T3 as well as the bioactive thyroid hormone metabolite TRIAC administration is also studied. The protocol for assessing the vestibular symptoms following the excitotoxic lesion includes a rating score and automated assessment of the posturo-locomotor symptoms constituting the syndrome. The follow-up study lasts 7 days after induction of the vestibulopathy, until recovery.

## Methods

### Animals and Ethics

All the applied procedures were previously approved by the ethics committee (MP-CEPAL n°22). The animals were housed in cages in social groups with enrichment of the environment. The accommodation room had a lighting cycle of 12 h:12 h and controlled temperature and humidity conditions. The animals were acclimatized to the experimenter and the experiment room for 5 days before the beginning of the experiments. The pre-defined endpoints were monitored throughout the study. A total of 146 adult male Long Evans rats (Janvier-labs) were included in the study, including 122 for the study of the effect of the administration of levothyroxine (114 after application of the exclusion criteria) and 24 for the study of the effect of the administration of metabolites.

### Experimental Design

**Figure 1:**
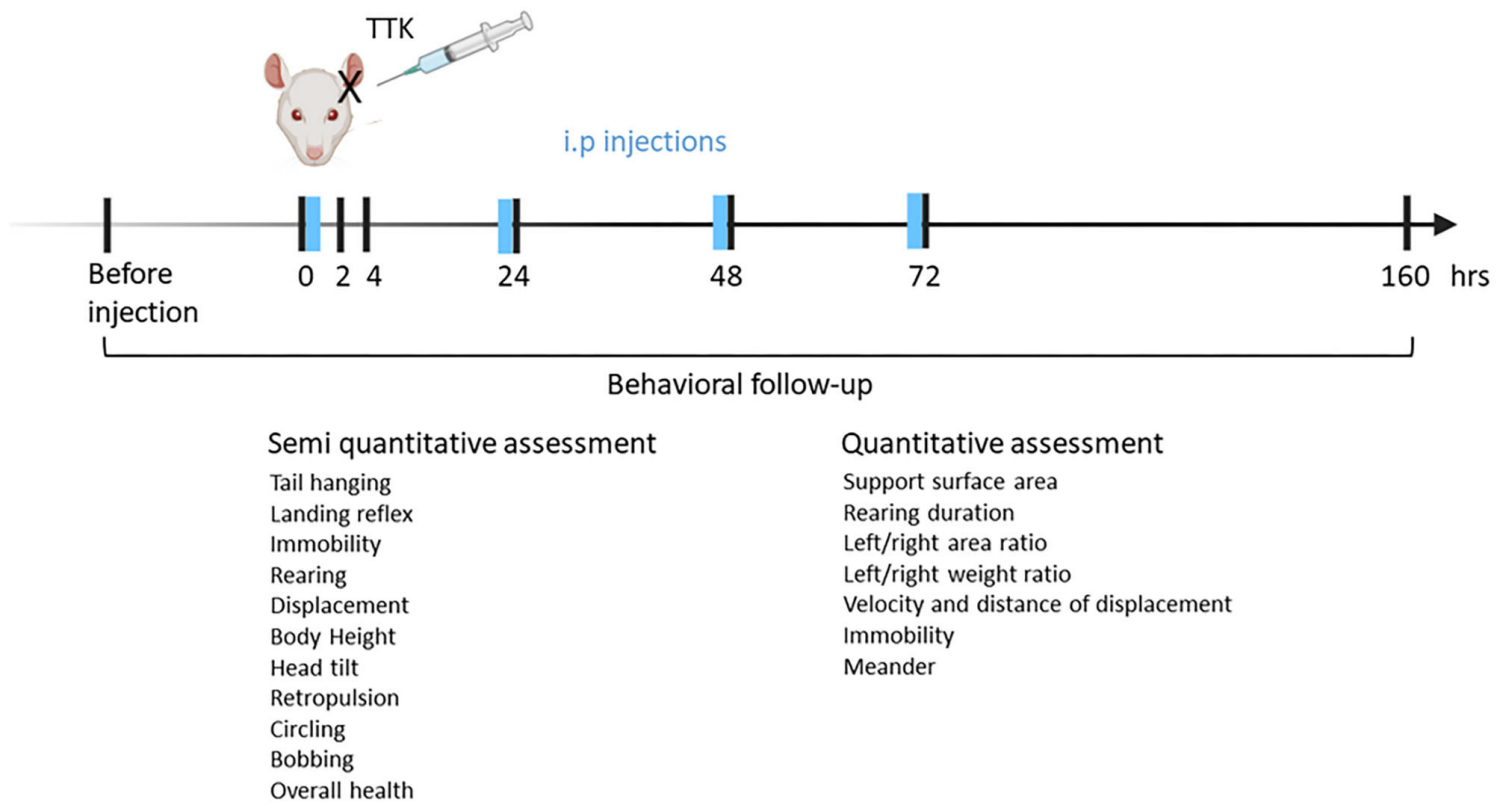
Experimental design. Vestibular excitotoxic injury is induced by subjecting anesthetized male adult rats to unilateral transtympanic injection of kainic. The animals are left to rest for the next 30 min on the ipsilateral side of the injection. T4 or T3 is immediately administered through intraperitoneal injection. To assess the effect of repeated T4 or T3 administration on the vestibular syndrome, the compound administration is repeated for the next 3 days before behavioral tests. Behavior follow-up is started before transtympanic injection for control values and continued at 2, 4, 24, 48, 72 h, and 1 week.

The follow-up study started the week before surgery for acclimatization to behavioral tests and collecting preoperative data and lasted until 1 week after the induction of the peripheral vestibulopathy (Figure 1). Treatment was injected intraperitoneally either once immediately after surgery or each day until 72 h (in blue). Both rating scores and automated evaluation were performed to assess the expression of the vestibular syndrome. Rats were euthanized at 1 week after the last test session.

### Induction of Peripheral Vestibulopathy

Induction of the excitotoxic-type vestibular injury was carried out by a transtympanic injection of 100 μL of 25 mM kainate (TTK) in 0.9% NaCl on a rat maintained under gas anesthesia with isoflurane (2–3%). A subcutaneous injection of 1 mL of sterile 0.9% NaCl prevented rats from dehydration. The rat was kept under anesthesia in a lateral position for additional 30 min. Then, before the animal wakes up, a first intraperitoneal injection of the compound was carried out. The animal was isolated for the first 24 h of its recovery. The general condition of the rat, the weight and the pre-defined endpoints were monitored throughout the operative process and post-operative follow-up.

### Treatment

Levothyroxine (T4, T2501, Sigma Aldrich, Gilligham, UK) was dissolved in sterile 0.9% NaCl solution and administered to rats by intraperitoneal injection at the desired dose. Solutions of triiodothyronine (T3, 64245, Sigma Aldrich, Gilligham, UK) and triiodothyroacetic acid (TRIAC, T7650, Sigma Aldrich, Gilligham, UK) were prepared and administered in the same way. The first phase of the project corresponded to the study of a daily administration of the T4 treatment over 4 days starting from the same day of the excitotoxically-induced vestibular injury. The second phase of the project corresponded to the study of a single administration of the T4 treatment immediately after the kainic acid transtympanic injection. The third phase of the project corresponded to the study of a daily administration of T3 alone or in combination with TRIAC over 4 days starting from the same day of the excitotoxically-induced vestibular injury. Doses of 10 μg/kg of T4 and T3 were based on previous study showing neuroprotective effect of thyroid hormones in rat models of vestibulopathy (8), and in rodent models of stroke (13, 14).

The following groups were composed according to the dose and chronicity of administration of the compound and were proceeded for behavioral tests randomly. For chronic administration of the compound, a first control group received repeated intraperitoneal injections of NaCl 0.9% following the transtympanic injection of kainic acid (G1, Saline, *n* = 20). Four treated groups (groups 2, 3, 4, and 8) received repeated intraperitoneal injections of T4 at different doses of 10, 5, 1, and.1 μg/kg (G2, T4_4x10, *n* = 23; G3, T4_4x5, *n* = 11; G4, T4_4x1, *n* = 12; G8, T4 4x0.1, *n* = 12). For single administration of the compound, three treated groups (groups 5–7) received a single T4 injection at different doses (G5, T4_1x10, *n* = 12; G6, T4_1x5μg/kg, *n* = 12; G7,T4 1x1, *n* = 12). For chronic administration of T3 and TRIAC, the two following groups (groups 9 and 10) received either T3 injection alone or in combination with TRIAC (G9, T3_4x10, *n* = 12 and G10, T3+TRIAC_1x10, *n* = 12). Indicated numbers take into account the exclusion of subjects after the application of the exclusion criteria below.

### Exclusion Criteria

Animals were excluded from the study in case of transtympanic injection failure (*n* = 2), hemorragy occurring during transtympanic injection (*n* = 1), under veterinary advice in case of complications such as respiratory or swallowing difficulties (*n* = 1), anxious behavior after transtympanic injections procedure not allowing to pass behavior test (*n* = 2), kainic acid poisoning expressed by trembling and convulsions within a few hours after transtympanic injections (*n* = 2).

### Behavioral Follow-Up of Vestibular Syndrome

The vestibular syndrome was assessed semi-quantitatively and quantitatively at postoperative times of 2, 4, 24, 48, 72 h, and 7 days. A preoperative test session was performed to collect the preoperative control values.

### Semi-Quantitative Evaluation

The vestibular syndrome was first stimulated by elevating the rat by the tail about twenty centimeters from the support in order to exacerbate the expression of symptoms. The vestibular syndrome was then assessed for 2 min according to a score scale established by Péricat et al. (15) and inspired by previous work from Zwergal group (Ludwig Maximilians University Munich, Germany).

Péricat et al. (15). A score of 0 (no symptoms) to 3 (severe symptom) was assigned to the following 11 criteria, for a maximum total score of 33: torsional movement of the body when the rat is lifted by the tail over the support (tail hanging behavior), landing reflex of the rat when dropped on the support (landing reflex), prostration time (immobility), ability to stand up on the hind legs (rearing), quality of gait and general movements of the animal (quality of displacement), body height and lift (body height), lateral tilt of the head (head tilt), reverse walk (retropulsion), concentric trajectories (circling), vertical repetitive movements of the head (bobbing) and general condition.

### Quantitative Evaluation

The impact of vestibular syndrome on the overall motor and exploratory behavior of the animal was assessed by the openfield test. The animals were accustomed to the device for 5 min per day for 3 days before the start of the experimental follow-up. Only values of the last preoperative session constitute the pre-operative values of reference. As for semi-quantitative assessment, the vestibular syndrome was first stimulated by elevating the rat by the tail about twenty centimeters from the support in order to exacerbate the expression of symptoms. The animal was placed in the center of the device and its movements were tracked and recorded by a camera located above the device for 5 min. The following parameters were calculated automatically by the Ethovision XT 15 software (Noldus, Wageningen, the Netherlands): Distance moved (m), mean velocity (cm/s), maximum velocity (cm/s), meander (°/cm), immobility time (s). Values were normalized as a percentage of pre-operative values for each animal, the preoperative value constituting 100%. For quantitative evaluation in Openfield, only part of the effective of groups 1 (*n* = 12) and 2 (*n* = 11) were used. Detailed assessment of posturo-locomotor symptoms of vestibular syndrome were assessed with a dynamic weight bearing device and analyzed using associated software (DWB 2, Bioseb). This device had previously been used to objectively and automatically characterize the vestibular syndrome in vestibular defective rats (16).

Seven parameters were analyzed across all postures. Then, using an immobility threshold (700 ms), postures were analyzed according to whether they were recorded under dynamic (<700 ms) or static conditions (>700 ms). The base of support of the animal body was evaluated by the parameter of the support surface area. The lateral distribution of the support surface was evaluated by the overall left/right area ratio of the four paws then more specifically by the left/right area ratio of the hind limbs. The lateral weight distribution was evaluated in the same way by the overall left/right weight distribution ratio and by the left/right weight distribution ratio applied to the rear legs. The animal's ability to stand up on its hind limbs was assessed by the rearing duration. Values were normalized as a percentage of the pre-operative values for each animal, the preoperative values constituting 100% (1 for ratios).

### Statistical Analyses

Statistical analyses were performed using Graphpad Prism 9 software (GraphPad Software, San Diego, US). To study the effect of treatment on the course of vestibular syndrome expression over time, all treated vestibular defective groups were compared to the untreated vestibular defective control group by an analysis of variance (Two-way ANOVA for repeated measures). To perform multiple inter-group comparisons, a *post-hoc* Tukey test was applied. To compare the postoperative values at each observation time to the preoperative values of each group, a *post-hoc* Dunnett test was applied. For the sake of readability, given the large number of groups, only the result of the multiple inter-group comparison is shown on graphs (effect of the treatment) and not the comparison of all the post-operative values with respect to the pre-operative values (time course of the syndrome over time for each group). To study the effect of dose and chronicity, statistical analyses were carried on within the treated groups only when a significant effect of treatment on the phenotype had been shown. To determine the effect of administration chronicity, a three-way analysis of variance (dose, administration chronicity and time) with Tukey's *post-hoc* test was applied. To determine the dose effect, a two-way analysis of variance (dose and time) was applied to the treated groups in each study phase (chronic administration phase and single treatment administration phase). To overcome interindividual differences in the posture-locomotor parameters of the animals, the data were normalized against the pre-operative values for each animal. Values are expressed as mean ± SEM. A significant difference is indicated by ^*^ if *P* < 0.05, ^**^ if *P* < 0.01, ^***^ if *P* < 0.001 and ^****^ if *P* < 0.0001.

## Results

### Effect of T4 Administration in a Model of Excitotoxically-Induced Vestibulopathy (TTK): Semi-Quantitative Assessment of the Vestibular Syndrome Expression and Assessment of Global Locomotor Impairment

Following the induction of the vestibular syndrome through TTK injections, the effect of T4 treatment on the course of symptoms was assessed from 2 h up to 1 week and data were compared to pre-operative' values (Figure 1).

**Figure 2:**
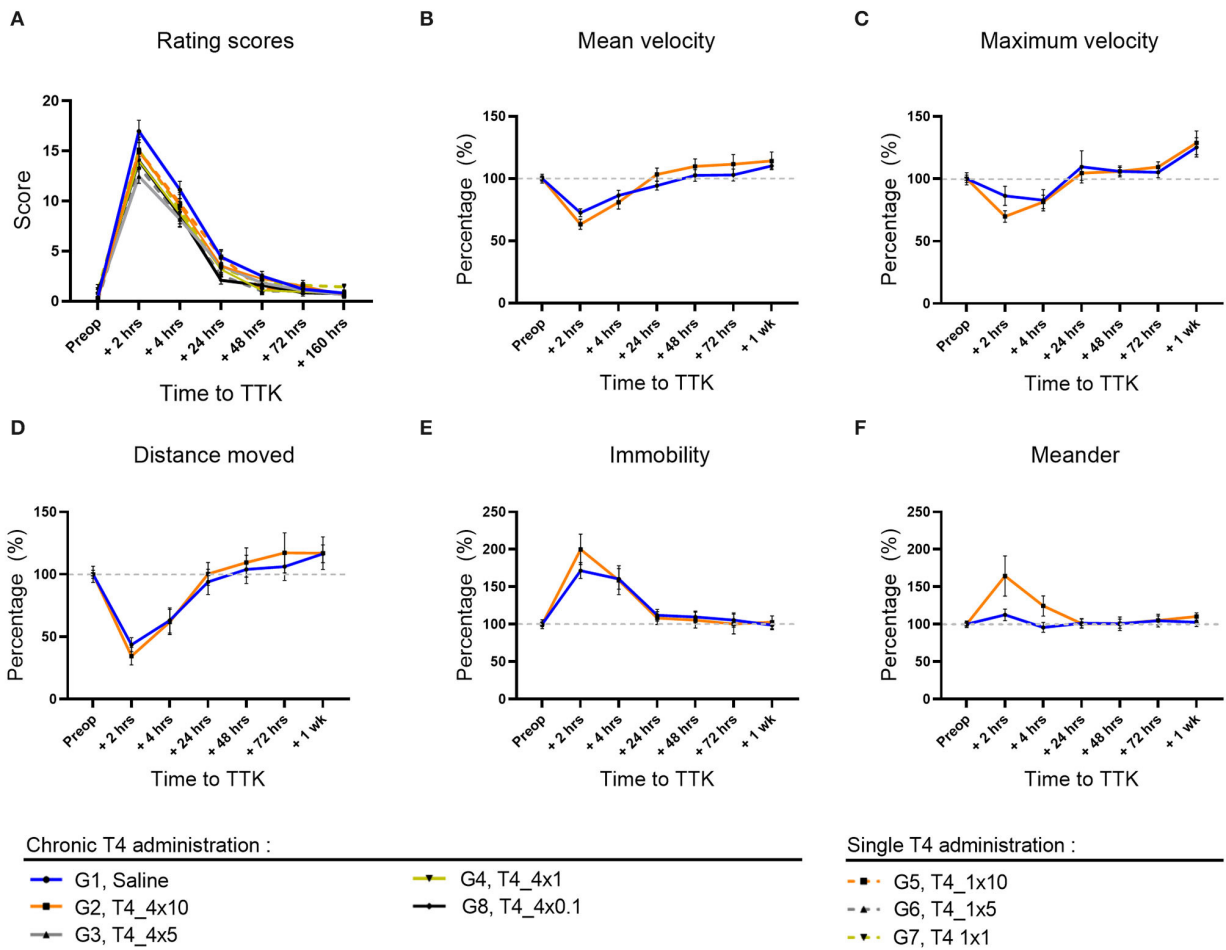
Effect of T4 treatment in a rat model of excitotoxically-induced vestibulopathy: semi-quantitative assessment of the vestibular syndrome and global assessment of locomotor function. **(A)** The vestibular syndrome reaches its peak value at 2 h post-TTK, having significant difference from preoperative values at 2 h (16.95 vs.35, *p* < 0.0001), 4 h (11.1 vs. 35, *p* < 0.0001), 24 h (4.4 vs. 35, *p* < 0.0001) and 48 h (2.5 vs. 35, *p* = 0.0002) after injury, before a gradual return to preoperative values at 72 h and 7 days. **(B–F)** Effects of T4 treatment on the indicated motor behavior in vestibular defective rats at the different time points. T4 treatment shows no significant effect on any of the considered parameters (Two-way ANOVA, NS). Semi-quantitative evaluation: *n* = 114. Openfield test: *n* = 12 TTK-Sham, *n* = 11 TTK-Treated T4.

First, the kinetics of expression of the induced vestibular syndrome was described by comparing the time course of the rating scores of untreated vestibular defective rats (G1, Saline) to their preoperative condition (Figure 2A). In comparison to the preoperative condition, untreated vestibular defective rats described a syndrome at its peak at 2 h post-injury TTK (16.95 vs. pre-op.35; *p* < 0.0001) and still significantly expressed at 4 h (11.1 vs. pre-op.35; *p* < 0.0001), 24 h (4.4 vs. pre-op.35, *p* < 0.0001) and 48 h (2.5 vs.35; *p* = 0.0002) post-injury before a gradual return to preoperative values at 72 h and 7 days. Then, the effect of T4 treatment was assessed semi-quantitatively by comparing the score of all T4-treated groups to the untreated vestibular defective group. The semi-quantitative assessment of vestibular syndrome did not show any significant effect of the T4 treatment on the excitotoxically-induced vestibular syndrome (Figure 2A).

The effect of T4 treatment on vestibular syndrome was further studied by automated measurement of the animals' speed of displacement, maximum acceleration, traveled distance, immobility duration and deviation from the trajectory (*meander*). The openfield test was performed on a group of 12 untreated vestibular defective animals and a group of 11 treated vestibular defective animals that received chronic T4 administration at a dose of 10 μg /kg. First, the effect of the TTK-induced vestibular syndrome was analyzed within each group.

The vestibular syndrome induced a significant decrease in the average speed of movement in all vestibular defective animals. In the untreated vestibular defective group, the mean speed reached its minimum at 2 h postoperatively (G1, Saline: 73%, *p* = 0.0036) (Figure 2B) and was no longer significant from 4 h. The average speed reached the preoperative threshold in the vicinity of 24 h (G1, Saline: 94%). The acceleration of displacement also decreased at 2 h but not in a significant way in the untreated vestibular defective group (G1, Saline: 86%) (Figure 2C). From 4 h, the decrease in acceleration was no longer significant and the acceleration approaches the preoperative values at 24 h (G1, Saline: 110%).

The decrease in velocity and acceleration of displacement induced by the vestibular syndrome was accompanied by a significant decrease in traveled distance in the untreated vestibular defective group at 2 h (G1, Saline: 44%; *p* = 0.0013) (Figure 2D). With regard to the velocity, the traveled distance approached control values at 24 h (G1, Saline: 94%).

The immobility duration followed the inverse time course compared to the traveled distance. At 2 h, the immobility duration increased significantly in the untreated vestibular defective group (G1, Saline: 172%; *p* = 0.0024) compared to the pre-operative values (Figure 2E). From 4 h, the immobility duration no longer differed significantly and gradually returned to preoperative values at 72 h and 1 week (G1, Saline, 72 h: 106%).

The TTK-induced vestibular syndrome also altered the gait by increasing the meander behavior slightly for the untreated control group (G1, Saline, 2 h: 113 %) (Figure 2F). From 24 h, the gait values returned to preoperative values (G1, Saline, 24 h: 101%).

The effect of T4 treatment on global locomotor impairment of the vestibular syndrome was then assessed by comparing the T4-treated group to the untreated vestibular defective group for each of the parameters above (Figures 2B–F). For speed and traveled distance, repeated-measures analysis showed non-significant results for both main effect of treatment and for the interaction of treatment with time (Figures 2B–E). For the meander, the interaction of treatment with time was significant but not the main effect of treatment (Figure 2F). Altogether, these results suggest that T4 treatment did not induce any significant difference between the treated and untreated groups for all the parameters studied in Openfield (Two-factor ANOVA, NS).

Taken together, the semi-quantitative assessment of vestibular syndrome and assessment of the global locomotor behavior did not reveal any significant effect of T4 treatment in a model of excitotoxically-induced vestibular injury.

### Effect of T4 Administration in a Model of Excitotoxically-Induced Vestibulopathy (TTK): Detailed Assessment of Posturo-Locomotor Parameters (Dynamic Weight Bearing Test)

To further study T4 treatment effects on the excitotoxically-induced vestibular syndrome, a detailed and automated assessment of posturo-locomotor parameters was carried out on untreated and treated vestibular defective animals. For each posturo-locomotor parameter of interest, analyses were performed taking all postures together before distinct analyses of dynamic postures and static postures.

### Support Surface Area

The support surface area reflects the postural stability, making this parameter accurate to assess postural balance impairment and the animal ability to overcome vestibular deficits. First, the effect of the TTK-induced vestibulopathy on this parameter was assessed in untreated vestibular defective animals.

**Figure 3:**
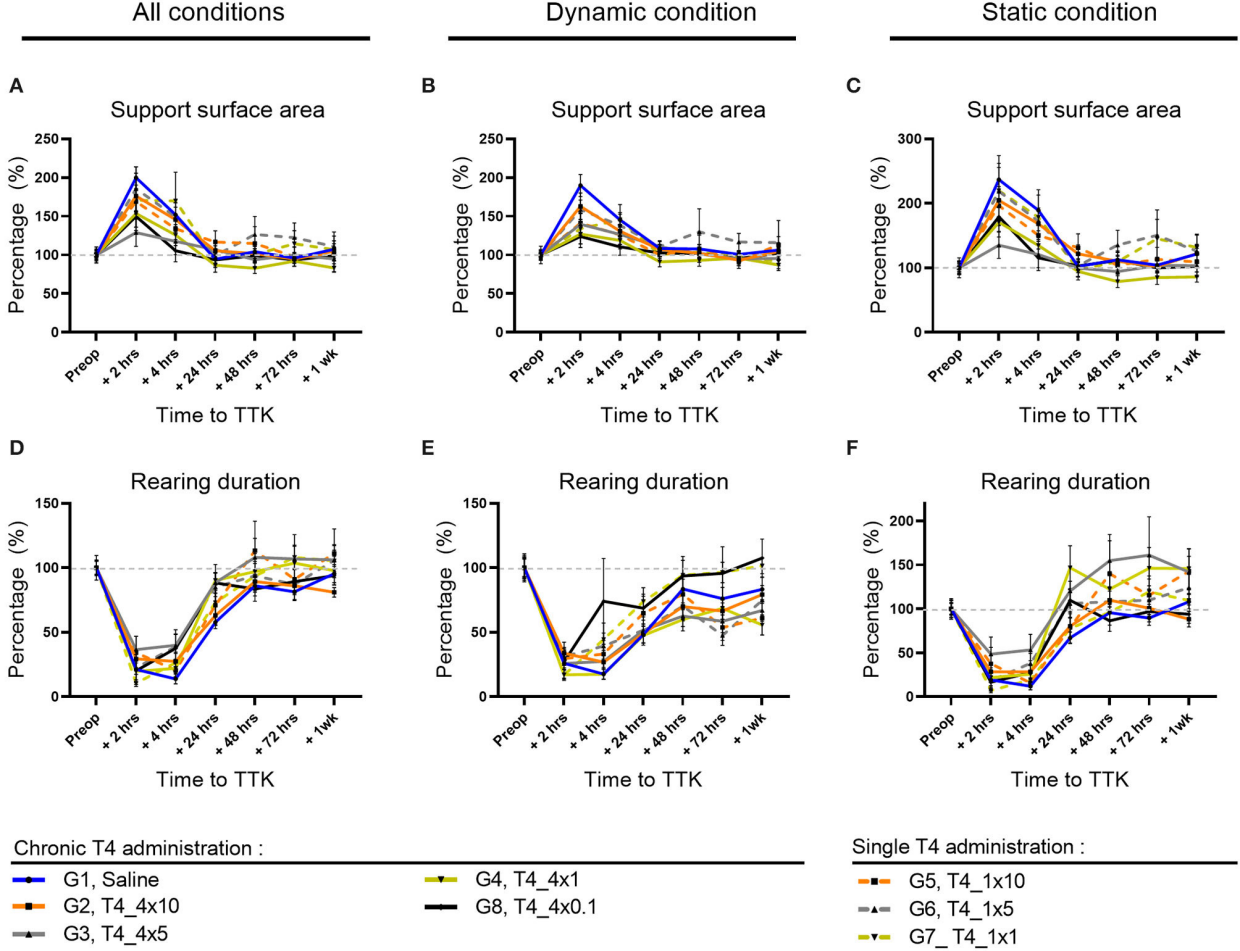
Effect of T4 treatment on support surface area and rearing ability in vestibular defective rats. **(A–C)** Support surface area. **(D–F)** Rearing ability. T4 treatment shows no significant effect on any of the considered parameters (Two-way ANOVA, NS). *n* = 114.

Altogether, untreated vestibular defective animals subjected to TTK showed an increased support surface area in untreated vestibular defective animals compared to the pre-operative data (Figures 3A–C). For all postures together, in comparison with the preoperative condition, the support surface area reached a significant peak at 2 h in untreated vestibular defective animals (G1, Saline, 2 h: 200% *p* < 0.0001) (Figure 3A). At 4 h, the increase in support surface area remained significantly greater than preoperative values in the untreated vestibular defective group (G1, Saline, 4 h: 152%; *p* = 0.0303). Beyond 24 h, the support surface area no longer significantly differed from the preoperative values and decreased until reaching the preoperative values from 48h (G1, Saline, 48 h: 104%).

Similarly, under dynamic conditions, the support surface was significantly increased up to 4 h post-TTK in the untreated vestibular defective group (G1, Saline, 2h: 190% *p* = 0.0001 and 4h: 145% *p* = 0.0026) (Figure 3B).

In the same way, under static conditions, the support surface area reached a significant peak for the untreated vestibular defective group (G1, Saline, 2 h: 237% *p* ≤ 0.0001) (Figure 3C).

The effect of the T4 treatment was then assessed by comparison with the untreated vestibular defective group. T4 treatment did not induce any significant difference between the treated and untreated groups for the support surface parameter (Two-way ANOVA, NS) (Figures 2A–C).

### Rearing Duration on the Hind Limbs

To better study, the postural equilibration of vestibular defective animals, the duration of rearing behaviors was automatically calculated over time (Figures 3D–F). First, the effect of the TTK-induced vestibulopathy on the animal rearing behavior was assessed in untreated vestibular defective animals. The vestibular syndrome induced a significant decrease in rearing duration in untreated vestibular defective rats up to 24 h under all conditions (Figure 3D) as in dynamic conditions (Figure 3E), and up to 4 h in static conditions (Figure 3F). Taking all postures together, the rearing duration dropped to 21% of the preoperative value (*p* = 0.0009) at 2 h, 14% at 4 h (*p* < 0.0001) and rose to 28% at 24 h (*p* = 0.0023) before a gradual return to preoperative values (Figure 3D). Under dynamic conditions, the rearing duration dropped to 26% at 2 h (*p* = 0.0051), 18% at 4 h (*p* < 0.0001) and rose to 48 % at 24 h (*p* = 0.0167) before gradually reaching the preoperative values without significantly differing (Figure 3E). Under static conditions, the rearing duration dropped to 19% at 2 h (*p* = 0.001) and 12% at 4 h (*p* < 0.0001) but increased rapidly to 68% of its value at 24 h and no longer differed from the preoperative values (Figure 3F).

The effect of T4 treatment on rearing ability was then assessed by comparison with the untreated vestibular defective control group. T4-treatment did not induce a significant difference in the time to upright posture on the hind legs (Two-factor ANOVA, NS).

### Distribution of Paw Contact Areas on the Lateral Axis

**Figure 4:**
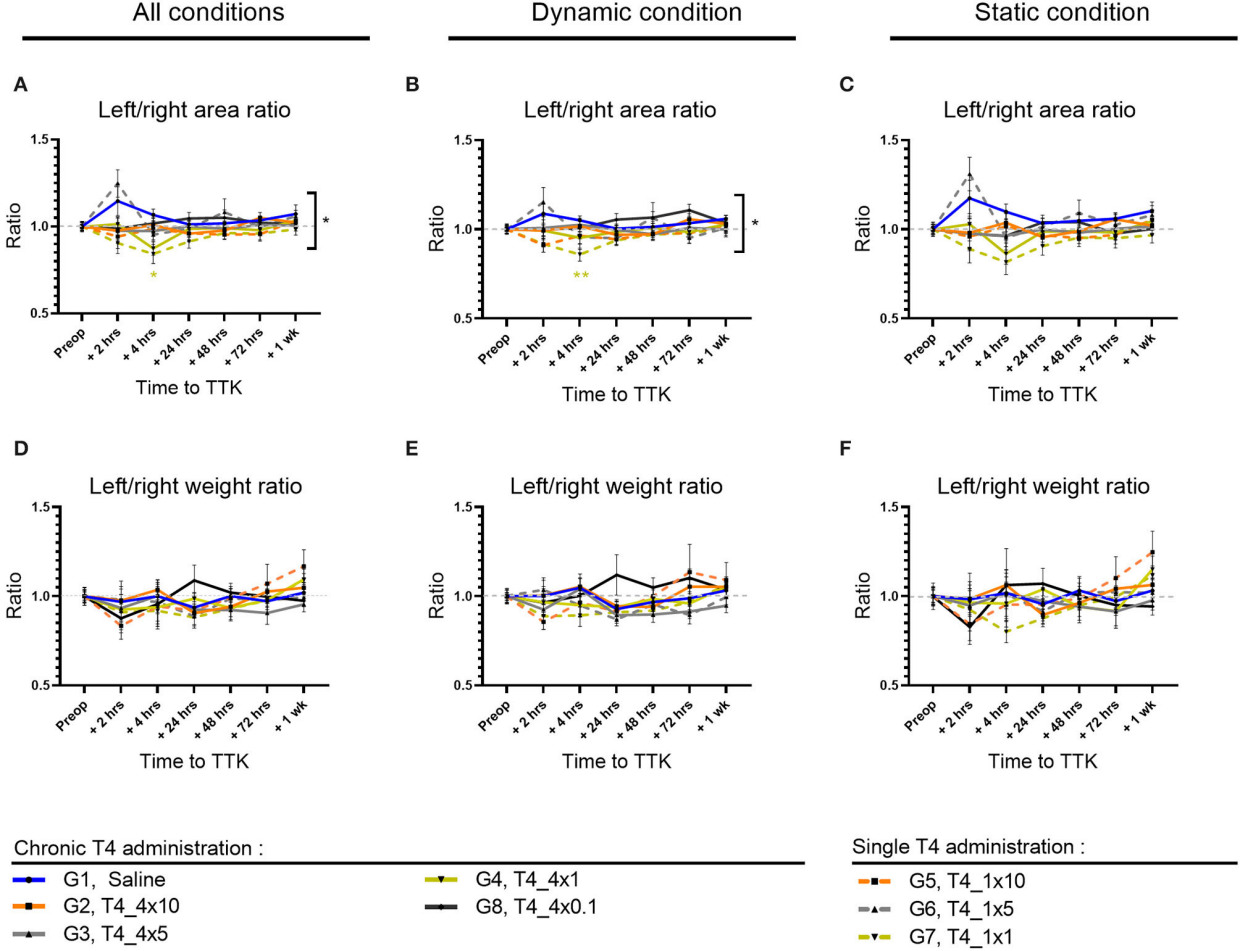
Effect of T4 treatment on left/right area **(A–C)** and weight **(D–F)** ratio in vestibular defective rats. Postural balance is assessed for all postures **(A,D)** or under dynamic **(B,E)** or static conditions **(C,F)**. Statistically significant differences are assessed using two-way Anova with Tukey *post-hoc*, *p < 0.05, **p < 0.01. *n* = 114.

To further study the postural imbalance of vestibular defective animals, the distribution of paw contact areas on the lateral axis was assessed by measuring paw print areas of all paws (Figures 4A–C) and specifically on the hind limbs (data not shown). First, the effect of the TTK-induced vestibulopathy on the distribution of paw contact areas was assessed in untreated vestibular defective animals. For all postures together, the vestibular syndrome induced a slight and non-significant alteration of the paw print areas distribution ratio on the lateral axis of untreated vestibular defective animals. The alteration of the paw print areas distribution was in favor of a transient displacement of the contact surface on the left side ipsilateral to the lesion at 2 h (G1, Saline, 2 h: 1.146 vs. preop: 1).

The effect of T4 treatment on the distribution of paw contact areas on the lateral axis was then assessed by comparing the values of all T4-treated groups to the untreated vestibular defective group. The T4 treatment induced a significant effect on the lateral distribution of paw print areas over time compared to untreated vestibular defective animals (Two-factor ANOVA with Tukey *post-hoc, p* = 0.018). For all postures together, T4-treated groups that received single or repeated doses of 1μg/kg of T4 differed significantly from the untreated injured control group at 4 h after TTK (G7, T4_1x1,.8417 vs. 1.067, *p* = 0.0401 and G4, T4_4x1,.8733 vs. 1.067, *p* = 0.00177) and showed a decrease in the left/right paw area ratio in favor of a support surface predominantly on the right contralateral side to the lesion (Figure 4A). Under dynamic conditions, the effect of the T4 treatment was significant (Two-factor ANOVA with Tukey *post-hoc, p* = 0.0294) more specifically for the T4-treated group with a single dose of 1 μg/kg (G7, T4_1x1,.8583 vs. 1.051, *p* = 0.0053) (Figure 4B). Finally, under static conditions, the same trend was observed for the T4-treated groups at a dose of 1 μg/kg without significantly differing (Two-factor ANOVA, *p* = 0.0913) (Figure 4C). In a second stage, the distribution of contact areas was specifically analyzed on hind paws. The left/right areas ratio of hind paws followed a similar trend to the distribution ratio including the four paws, both on all the postures, under dynamic conditions and static conditions (data not shown). However, the treatment did not show any significant interaction with the course of this parameter over time on hind limbs.

To investigate the effect of the chronicity of treatment administration on the lateral distribution of paw print areas, statistical analysis taking into account the dose and frequency of administration over time was carried out for all of the treated groups (with the exception of the group treated with the.1 μg/kg dose which was treated only chronically). However, the study did not show any significant effect of chronicity of treatment administration on this parameter.

Then, the dose-effect was more specifically assessed within T4-treated groups, either within chronically treated groups or within once treated groups. The chronically T4-treated groups did not offer evidence of a significant dose-effect on this parameter. However, for all postures combined, the single T4-treated groups differed significantly between 10, 5, and 1 μg/kg doses at 2 h postoperatively (*p* = 0.0197): the 10μg/kg dose differed significantly from the 5 μg/kg dose (G5,T4_1x10, 2 h:.9417 vs. G6,T4_1x5: 1.248; *p* = 0.0196) and the 5μg/kg dose also differed significantly from the dose 1μg/kg (G6,T4_1x5, 2 h: 1.248 and G7,T4_1x1, 2 h:.9042; *p* = 0.0061). The single T4-treated groups with doses of 10μg/kg and 1μg/kg showed values closer to the pre-operative value than the dose of 5μg/kg.

Altogether, analyses of the distribution of paw contact areas on the lateral axis show that a single 1 μg/kg T4 dose is the only dose of treatment that differs significantly both by comparison with the untreated group and within treated groups.

### Weight Distribution on the Lateral Axis

To study the weight balance distribution of vestibular defective animals, the weight distribution ratio was automatically calculated over time. First, the effect of the TTK-induced vestibulopathy on the weight balance distribution was assessed in untreated vestibular defective animals. The TTK excitotoxically-induced vestibular syndrome did not alter the lateral weight distribution ratio automatically calculated on all the supports of the untreated vestibular defective animals (Figures 4D–F), both when considering all the postures (G1, Saline, 2 h:.97 vs preop: 1) (Figure 4D) under dynamic conditions (G1, Saline, 2h: 1 vs. preop: 1) (Figure 4E) and under static conditions (G1, Saline, 2 h:.98 vs. preop: 1) (Figure 4F). The weight distribution ratio on hind limbs followed the same trend for all postures (G1, Saline, 2 h:.96 vs. preop: 1) both in dynamic (G1, Saline, 2 h: 1.036 vs. preop: 1) and static conditions (G1, Saline, 2 h:.94 vs. preop: 1) (data not shown).

The effect of T4-treatment on the lateral weight distribution was then assessed by comparing the values of all T4-treated groups to untreated injured animals. The T4 treatment did not induce any significant effect on the weight distribution ratio on the left/right lateral axis (Figures 4D–F).

Taken together, these results showed that T4 treatment in the TTK model did not have a significant effect on the lateral axis weight distribution ratio (Figures 4D–F).

### Effect of T3 and TRIAC Administration in a TTK Model of Excitotoxically-Induced Vestibulopathy: Assessment of Vestibular Syndrome in Vestibular Defective Animals Treated With T3 and TRIAC

**Figure 5:**
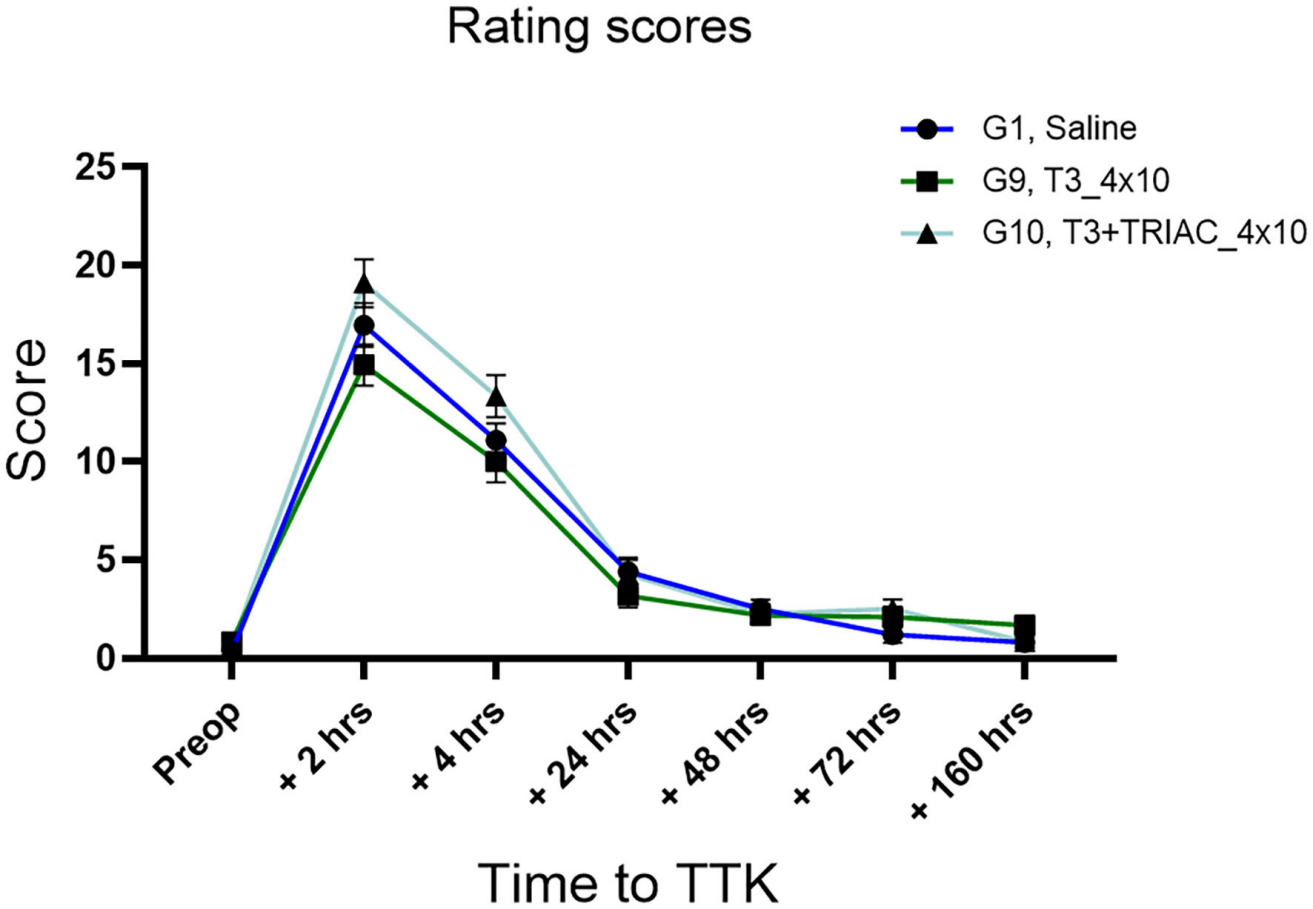
Semi-quantitative assessment of the T3 treatment on vertigo syndrome in vestibular defective rats. T3 treatment shows no significant effect on the considered parameters (Two-way ANOVA, NS). *n* = 44.

The effect of chronic administration of triiodothyronine and triiodothyroacetic acid to vestibular defective animals was first assessed by rating scores (Figure 5). First, the effect of the TTK-induced vestibulopathy was assessed by comparing post-operative and pre-operative conditions. After induction of the TTK model, the T3-treated group described a vestibular syndrome that significantly differed from their preoperative values up to 24 h (at 2 h: 14.92 vs. preop.8333 *p* < 0.0001, at 4 h: 10 vs. 8333, *p* < 0.0001 and at 24 h: 3.167 *p* = 0.0109) but not at 48 h (at 48 h: 2.167, *p* = 0.0546, NS). Vestibular defective animals treated with T3 in combination with TRIAC showed a significant vestibular score up to 24 h (at 2 h: 19.08, *p* < 0.0001 at 4h 13.33 *p* < 0.0001 at 24 h 4.250 *p* = 0.0172 vs. preop.6667) and at 72 h (at 72 h: 2,500 *p* = 0.0289) (Figure 5).

The effect of the T3 treatment with or without the combination of TRIAC on the course of the vestibular syndrome was then assessed by comparing their rating scores from the untreated vestibular defective animals. The semi-quantitative assessment of the syndrome did not show any significant effect of the T3 treatment alone or in combination with TRIAC on the course of the vestibular syndrome (Figure 5).

### Effect of T3 and TRIAC Administration in a TTK Model of Excitotoxically-Induced Vestibulopathy: Detailed Assessment of Posture-Locomotor Parameters in Vestibular Defective Animals

**Figure 6:**
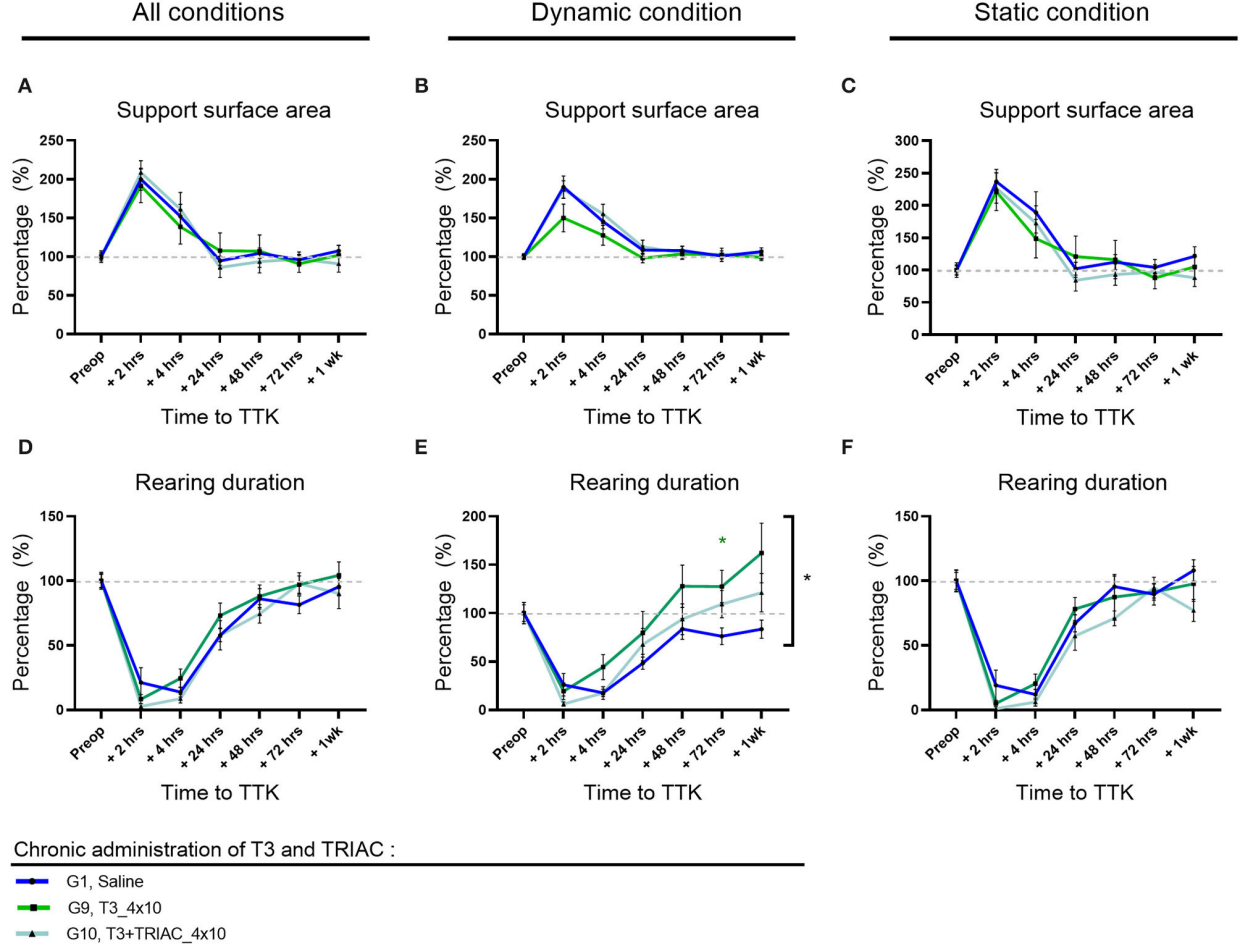
Effect of T3 alone or a combination of T3+TRIAC treatments on the support surface area **(A–C)** and rearing duration **(D–F)** in vestibular defective rats. Statistically significant differences are assessed using two-way ANOVA with Tukey *post-hoc*, *p < 0.05. *n* = 44.

To study the effect of T3 treatment alone or in combination with TRIAC on the syndrome, a detailed and automated assessment of posturo-locomotor parameters was carried out as previously. For each parameter of interest, the analysis was carried out on all the postures (Figures 6A,D) then on the dynamic postures on the one hand (Figures 6B,E), and static on the other hand (Figures 6C,F).

### Support Surface Area

As previously described, the vestibular syndrome induced a significant increase in the surface of the support polygon in untreated vestibular defective animals at 2 h and 4 h postoperative (Figure 6A). This significant alteration persisted for up to 4 h for these control animals under dynamic conditions (Figure 6B) and up to 2 h under static conditions (Figure 6C). For all postures together, animals treated with T3 alone and in combination with TRIAC exhibited a support polygon that differed significantly from their preoperative values only at 2 h (G9, T3, 2 h: 191%, *p* = 0.0179 and G10, T3+TRIAC, 2 h: 209% *p* = 0.0008) (Figure 6A). Likewise, under static conditions, animals treated with T3 alone or in combination with TRIAC differed significantly from their preoperative values only at 2 h (G9, T3, 2 h: 221%, *p* = 0.0242 and G10, T3+TRIAC, 2 h: 227% *p* = 0.0107) (Figure 6C). Under dynamic conditions, however, only the T3 treated group in combination with TRIAC significantly differed from its preoperative values up to 4 h (G10, T3+TRIAC, 2 h: 187% *p* = 0.0001 and 4 h 155% *p* = 0.0073) while the treated group that received T3 alone did not differ significantly from its preoperative values (Figure 6B).

The effect of treatment with T3 alone or in combination with TRIAC was then assessed by comparison with the untreated vestibular defective group (Figures 6A–C). Treatment with T3 alone or in combination with TRIAC did not induce a significant difference (Two-factor Anova, NS).

### Rearing Duration on the Hind Limbs

The rearing duration of vestibular defective animals was automatically calculated over time (Figures 6D–F). As previously described, the vestibular syndrome induced a significant decrease in rearing duration in untreated vestibular defective animals up to 24 h under all conditions (Figure 6D) as in dynamic conditions (Figure 6E), and up to 4 h in static conditions (Figure 6F). The effect of T3 treatment alone or in combination with TRIAC on rearing duration was assessed by comparison with the untreated vestibular defective group. T3 treatment alone showed a significant effect on rearing duration at 72 h compared to the TTK-Sham control group in dynamic conditions only (Figure 6E) (G9, T3, 72 h: 127% *p* = 0.036) and did not differ in static conditions (Figure 6F) or all postures together (Figure 6A).

Taken together, the results of this quantitative analysis did not show a significant effect of T3 treatment alone or in combination with TRIAC on the course of vestibular syndrome, with the exception of a significantly increased rearing duration at 72 h after TTK.

### Distribution of Paw Contact Areas on the Lateral Axis

**Figure 7:**
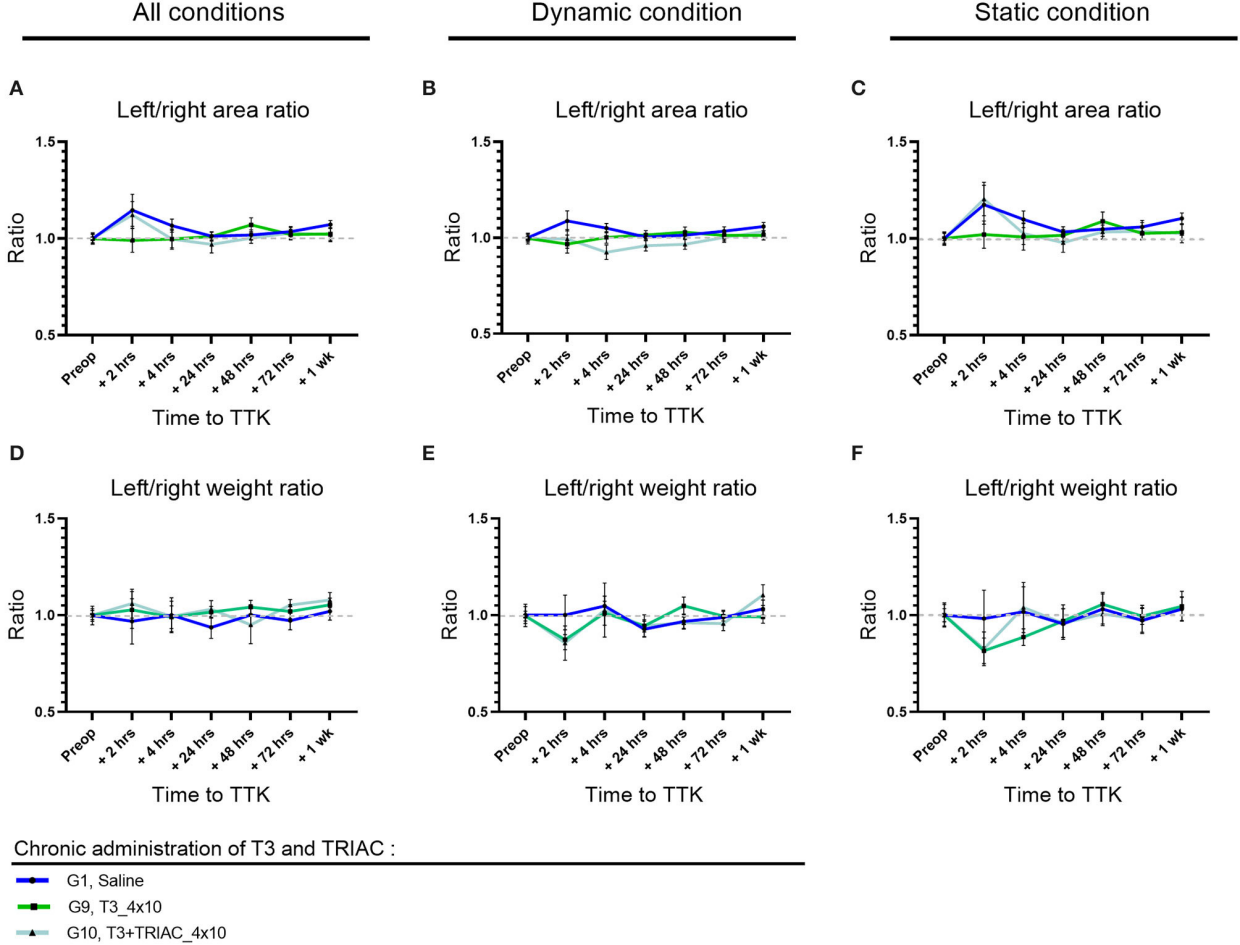
Effect of T3 treatment on left/right area **(A–C)** and weight **(D–F)** ratio in vestibular defective rats. Postural balance is assessed for all conditions **(A,D)** or under dynamic **(B,E)** or static conditions **(C,F)**. T3 treatment shows no significant effect on the considered parameters (Two-way ANOVA, NS). *n* = 44.

The impact of the vestibular syndrome on the distribution of the paw contact areas on the lateral axis was assessed by measuring paw print areas of all paws (Figures 7A–C) and specifically on the hind limbs (data not shown). The effect of T3 treatment alone or in combination with TRIAC was assessed by comparison with the untreated vestibular defective group (Figures 7A–C) for all postures together (Figure 7A) and then under dynamic (Figure 7B) and static (Figure 7C) conditions. Treatment with T3 alone or in combination with TRIAC did not show any significant effect on the paw contact areas on the lateral axis (Two-factor Anova, NS).

### Weight Distribution on the Lateral Axis

As previously described, the TTK model of excitotoxically-induced vestibular syndrome did not significantly alter the overall weight distribution ratio (Figures 7D–F) or hind limbs (data not shown) in untreated vestibular effective animals. Like the T4 treatment, the T3 treatment alone or in combination with TRIAC did not induce any significant alteration in the overall left/right weight distribution ratio (Figures 7D–F) and in the hind limbs (data not shown) (Two-factor Anova, NS).

## Discussion

### Partial and Reversible Unilateral Deafferentation Induced by Transtympanic Administration of Kainic Acid: A Model Comparable to the Clinical Manifestations of the Acute Peripheral Vestibulopathy Disorder

Evidence of an antivertigo effect of T4 was previously shown in a model of vestibular disorder-induced through a unilateral surgical section of the UVN (8). The experimental model of vestibulopathy used in the current study differs from the UVN model in several aspects.

First, it has previously been shown that TTK induces an excitotoxic lesion which causes deafferentation of hair cells in the inner ear (10, 17, 18). Between 2/3 and 3/4 of synaptic contacts are altered by this procedure (12). Therefore, a small proportion of the contacts between hair cells and fibers of the vestibular nerve are preserved. Conversely, UVN induces a total loss of inputs from the peripheral vestibular sensors on the ipsilateral side of the injury (15, 19). A second major difference is the reconstitution of synaptic contacts between hair cells and nerve fibers within a few days in the TTK model, in contrast to the irreversibility of the UVN model where no peripheral repair occurs. Third, UVN induces complete loss of the inputs arising from both the vestibular endorgans and the Scarpa ganglion neurons. This section causes Wallerian degeneration of fibers that project into the VN of the brainstem. This degeneration leads to a mosaic of plastic events that occur in the h and days after the section of the nerve, within the VN, and which are the original cause of the process of central compensation (20, 21). In addition, the kinetics of the vestibular syndrome produced by the TTK model is very different from that of the UVN model. While the time to reach the peak of the syndrome is comparable in both models, the duration of the phase of paroxysm of the symptoms lasts only 2 to 4 h at most for the TTK model, while it is extended over 3 to 4 days for the UNV model (15, 18, 22). Total resorption of the syndrome under the action of the central compensation process takes place in 48 h for the TTK model (12, 18), while it lasts over several weeks for the UVN model (16, 22, 23). These semi-quantitative observations are generally found in the automated analyzes of the various posturo-locomotor parameters studied.

In the current study, rats subjected to an excitotoxically-induced vestibular injury show a sudden and intense expression of vestibular syndrome which reaches its peak 2 h after the lesion, gradually decreases during the first 48 h, and finally returns to preoperative values at 72 h. This expression kinetics of the syndrome and its intensity are consistent with previous studies in the rat TTK model (18).

The locomotor function impairment was confirmed by an automated assessment of motor and exploratory behavior which showed locomotor function impairment during the first 24 h following injury. Thus, the rating scores of vertigo syndrome and the automated assessment of locomotor function are complementary and provide a basis for an exhaustive assessment of post-injury functional recovery. The macroscopic assessment of locomotor behavior of animals subjected to excitotoxic vestibular injury was supplemented by a detailed and automated assessment of weight distribution and support surface described as two postural stability parameters. The impact of vestibular function impairment on detailed posturo-locomotor parameters has been demonstrated previously in the rodent UVN model (16, 22, 24). While control healthy rats distribute their weight equally between the left and the right part of their body, UVN rats distribute more weight on the intact side (first-week post-UVN) than the lesioned side and then distributed more weight on the left side (from 7 to 30 days after injury). In contrast, in the TTK model used in the current study, postural asymmetry on the lateral axis is not significantly altered, either in the ratio of the lateral support areas distribution (left/right area ratio) or in the ratio of lateral weight distribution (left/right weight ratio). However, in the untreated vestibular injured group, the ratio of the lateral support areas distribution shows a trend to increase the paws areas on the ipsilateral side at 2 h post excitotoxic vestibular injury. In summary, the TTK model differs greatly from the UVN model in terms of the kinetics of syndrome expression, this model does not alter the weight distribution on the lateral axis and induces only a slight effect on the contact areas of the legs with the ground. The kinetic characteristics of the TTK model bring this model closer to the acute crisis encountered in Menière's disease and neuritis and offer a more accurate basis to assess potential therapeutic candidates for these diseases.

Precise etiological mechanisms leading to peripheral vestibulopathies have not been fully established so far. However, the excitotoxic deafferentation (TTK) model has been reported to induce partial deafferentation of peripheral sensors unilaterally (12). It can therefore be compared to the situations encountered in acute peripheral vestibulopathy (19). Conversely, the UVN model consists of a sudden and complete surgical section of the vestibular nerve unilaterally (15). It, therefore, produces the neurophysiological conditions encountered in vestibular neurotomy (25). Although it is currently unknown to what extent this peripheral damage impacts the central vestibular networks, our intention in the present study was to compare the effect of T4 administration on the vestibular syndrome evoked in both models. We measured in vestibular defective rats static and dynamic parameters similar to what is done in clinical tests. The support surface area parameter, assessed with the DWB device, has been analyzed in both static and dynamic conditions, in the same way as in posturographic tests to analyze posture in either static or dynamic conditions in humans. Dynamic tests were performed using videotracking. Some parameters such as the measure of walk trajectory (meander) can be extrapolated to the Fukuda test currently used in the clinic.

### Thyroxine Administration Modifies the Support Areas Distribution on the Lateral Axis but Does Not Significantly Improve Overall Motor Recovery in the Excitotoxic Vestibular Model

In the current study, T4 administration to rats that experienced unilateral TTK lesions did not prevent the alteration of the overall motor and exploratory behavior. Altogether, T4 treated and untreated injured animals showed similar locomotor deficits in terms of speed, acceleration, distance traveled, and immobility duration. In the same way, rating scores of vestibular syndromes do not show any significant effect of T4 treatment in vestibular injured animals, even if the untreated TTK group showed a trend to reach higher vestibular scores than TTK T4 treated group. The T4 administration did not show any significant effect on posturo-locomotor parameters. However, it is interesting to note the tendency of all TTK T4 treated groups to show a lesser enlargement of their support surface area conversely to the untreated TTK group (Figure 2B: *p* = 0.057), although this effect is not robust enough to be significant in the current model of partial and reversible vestibular injury. Thus, it cannot totally be ruled out that T4 treatment may promote the recovery of postural stability under more permissive conditions.

Secondly, the current study focused on establishing the optimum administration window and doses of T4 treatment. Given the shorter half-life of thyroxine in rats (12–24 h) compared to humans (5–9 days) (26), the treatment was administered intraperitoneally to rats either once immediately after TTK injection or for 4 days post-TTK including the day of the injury. Semi-quantitative and automated analyses did not show a difference in efficacy between single or repetitive administration of thyroxine in the TTK model. This suggests that a single administration of T4 is sufficient to induce the observed beneficial effect. Recently, a significant antivertigo effect of T4 administration in a model of a full section of the vestibular nerve (UVN) was demonstrated for an acute administration (1 injection for 4 days beginning immediately after UVN) of the compound at a dose of 10 μg/kg (8). In the present TTK model, T4 administration with the same dosage and route of administration (10 μg/kg *i.p*) did not produce any significant antivertigo effect on the overall locomotor behavior. Then, the T4 treatment was administered at doses of 5, 1, and 0.1 μg/kg. Detailed analysis of posturo-locomotor parameters suggests a greater effect in preventing postural alterations for the 1 μg/kg dose. This may be explained by the fact that administering high doses of exogenous thyroid hormones may activate a negative feedback mechanism of the hypothalamic-pituitary-thyroid axis. In fact, the T4 hormone, like T3, plays a role in the regulatory mechanisms of the hypothalamic-pituitary-thyroid axis and the negative feedback control of the level of circulating thyroid hormones (27–30).

### Administration of Triiodothyronine (T3), but Not Thyroxine (T4), Improves Equilibration Capacity Following Vestibular Injury

In order to study whether the antivertigo effect observed with T4 could be mediated by its conversion into active T3, we checked whether the direct administration of T3 alone or in combination with its metabolite TRIAC, affected the TTK induced vestibular syndrome. Rating scores of vestibular syndrome did not show any significant effect of treatment, although the TTK T3 treated group tends to show lower vestibular scores than TTK untreated animals. Unlike T4 treatment, T3 treatment alone or in combination with TRIAC did not show any significant effect, even slight, on the distribution of support surface areas. In contrast, T3 treatment alone showed a significant effect on the rearing duration of animals 72 h after vestibular injury. In comparison with the early T4 effect on the distribution of paw print areas (4 h post-TTK), the effect of T3 on the rearing ability of rats is more delayed (72 h). Assuming that the effect of T4 would first require its conversion to active T3, a faster antivertigo effect was expected after direct administration of T3. These results may be explained by the hypothesis that effects on postural symmetry (left/right area ratio) observed quickly at 4 h following the administration of T4 might depend on a rapid non-genomic action of T4 whereas the effects on the equilibration following the administration of T3 (rearing duration) of the animal at 72 h would depend on the slower genomic action of T3.

The effects of thyroid hormones can be mediated through genomic and non-genomic cellular pathways. Regarding non-genomic action, T4 and T3 both act on the membrane integrin receptor which, however, has a stronger affinity for T4 (31–33). T4 can then mediate its effects as a hormone or pro-hormone (31). Regarding genomic action, nuclear receptors show a markedly higher affinity for T3 than for T4, suggesting that T4 is used as a prohormone converted to active T3 by a deiodinase (31). Deiodinases play an important role in regulating the action of thyroid hormones which they can activate or deactivate, and the action of thyroid hormones is therefore partly dependent on the expression of deiodinases in different tissues (29, 34). The expression of the DIO2 enzyme that converts T4–T3 has been evidenced in tanycytes, astrocytic cells, and certain sensory neurons (35) and its expression has recently been shown for the first time in the vestibular nuclei (8).

Our results also show that a combined treatment of T3 plus TRIAC showed no effect on post TTK syndrome. Given the fact that TRIAC and T3 have an agonist action, at least the same effect as that induced by T3 administration might have been expected when T3 was combined with TRIAC. A first hypothesis relates to the fact that both T3 and TRIAC bind to the same receptors. TRIAC is a T3 metabolite that is described as exhibiting an agonist action on nuclear receptors for thyroid hormones. However, TRIAC differs from T3 by a higher affinity for the TRβ receptor than T3 and by a different transport mechanism which makes its use interesting for studies relating to the phenomenon of resistance to thyroid hormones (36, 37). A second hypothesis to explain that the effects of T3 are not enhanced by the addition of TRIAC is that TRIAC has a stronger impact than T3 or T4 on the mechanism of negative regulation of the thyroid axis, especially on TSH suppression (38, 39). Previous studies showed that the administration of TRIAC at a dose of 10 μg/kg in rats leads to a reduction in TSH within 6 h after its administration (40). Finally, the last hypothesis relates to the shorter half-life of TRIAC compared to T3, which might be too short to mediate notable effects in this current excitotoxic vestibular injury model (around 1 hour for TRIAC and 2 h for T3 in rats) (40, 41).

### Thyroxine Treatment Is More Effective in the Vestibulopathy Model Generated by the Unilateral Section of the Vestibular Nerve

In order to better understand the mechanism of action of T4 in the context of vestibulopathy and to better target its potential therapeutic indication, it is worth comparing the slight antivertigo effect observed in the present TTK model to the robust effect previously described in the UVN model (8). The surgical section of the vestibular nerve leads to sudden, complete, and irreversible loss of the peripheral vestibular inputs (15, 22, 42). In this model, T4 treatment improved both overall locomotor behavior (distance traveled, average speed and acceleration, time of immobility and deviation from the trajectory), weight distribution on the lateral axis, and support surface area (8). Conversely, in the TTK model, T4 and T3 treatments in combination or not with TRIAC, do not evoke a robust antivertigo effect on locomotor behavior.

The first hypothesis to explain these different antivertigo effects of T4 on the UVN and TTK models is based on the nature of the vestibular lesion which generates the expression of different reactive mechanisms in the vestibular nuclei (43–46). The first mechanism relates to the conversion of T4 to active T3 in the vestibular nuclei. In the CNS, the DIO2 enzyme which converts prohormone T4 into active T3 is synthesized in tanycytes, astrocytic cells, and certain sensory neurons (35). Its expression in the vestibular nuclei has been recently demonstrated [Rastoldo et al. (8)]. In the UVN model, the proliferation of microglial cells, astrocytes, and oligodendrocytes has been described in deafferented VN in several animal species including rats (9, 22, 43–46). Thus, an increased astrocyte population following UVN may support the increase of T4–T3 conversion, therefore potentiating the antivertigo effect of T4 administration. Unlike the UVN which results in Wallerian degeneration of the vestibular afferents projecting to the vestibular nuclei, it is likely that the peripheral excitotoxic insult does not evoke the strong glial proliferation in the VN on the injected site and that the conversion rate of T4–T3 may be not significantly altered.

A second hypothesis concerns a positive regulation of the TRα receptors in the deafferented VN in the UVN model (8) which could increase the binding of T4 and T3 and therefore their antivertigo effect. This process may not occur in the TTK model.

A third hypothesis to explain the differential antivertigo effect of T4 on the UVN and TTK models relates to the different expression kinetics of the vestibular syndrome generated in the two models. The UVN model consists of a full section of the vestibular nerve, inducing robust dizziness, peaking at 24 h and lasting for up to 30 days after injury. The TTK model leads to a lighter and shorter vestibular syndrome, exhibiting transient and reversible kinetics with a peak occurring within a few h. However, this brief kinetics of syndrome expression leaves a relatively short observation window for the assessment of the therapeutic effect of tested compounds. Similarly, it is more difficult to observe a pharmacological effect on a vestibular syndrome of low intensity.

Thus, the excitotoxic unilateral vestibular injury model might be more suitable for studying therapeutic compounds with a rapid mode of action and effect on the vestibular syndrome. Since the action of thyroid hormones can be non-genomic (a few minutes) or genomic (a few hours) (32), it is possible that the kinetics of the TTK model is too short compared to the UVN model to allow observation of a long-term effect of these hormones.

The difference in the underlying mechanisms of the UVN and TTK models makes these models accurate for studying different types of vestibular disorders. The UVN model reproduces the expression of a syndrome similar to that evoked following a surgical section of the vestibular nerve, as can be observed following Schwanoma surgery, while the TTK model reproduces the expression of a similar syndrome to that observed clinically during an acute vertigo attack as observed in acute peripheral vestibulopathy or in Ménière's disease, which makes it an appropriate model for testing potential therapeutic candidates on these pathologies.

### Limitation of the Study

The test battery used in the present study includes measures that are highly specific to vestibular dysfunction while some other behaviors are less specific. Taking into account the efforts to reduce to a minimum the use of animals, no sham group (with no kainic acid) was included in the experimental design to represent what is due to surgical procedure alone. Thus, we are aware that caution should be used in interpreting them. Another limitation of this study is that the present study was performed only on male rats. However, considering the translational context of this research and the fact that females are more likely than males to display vestibular pathology, the next step should be to investigate the effect of T4 administration in female vestibular defective rats.

## Data Availability Statement

The raw data supporting the conclusions of this article will be made available by the authors, without undue reservation.

## Ethics Statement

The animal study was reviewed and approved by MP-CEPAL n°22.

## Author Contributions

CC and BT conceived the study. CB performed behavioral pharmacology. CB performed the TTK injection. CB, BH, RB, CC, and BT contributed to data analysis and interpretation of the results. CB, CC, and BT wrote the manuscript, and all authors contributed to its editing. All authors contributed to the article and approved the submitted version.

## Funding

The project was supported by Vertidiag under the French Tech Emergence Grant from BPIFrance.

## Conflict of Interest

CB, BH, and RB were employed by Vertidiag. The study was made in affiliation with the Vertidiag company. The authors declare that this study received funding from Vertidiag. The funder had the following involvement in the study: collection, analysis, interpretation of data, the writing of this article and the decision to submit it for publication.

## Publisher's Note

All claims expressed in this article are solely those of the authors and do not necessarily represent those of their affiliated organizations, or those of the publisher, the editors and the reviewers. Any product that may be evaluated in this article, or claim that may be made by its manufacturer, is not guaranteed or endorsed by the publisher.
